# Evaluation of Pediatric Immediate Life Support Courses by the Students

**DOI:** 10.3390/children9020229

**Published:** 2022-02-09

**Authors:** Ignacio Manrique, Custodio Calvo, Angel Carrillo, Valero Sebastián, Gema Manrique, Jesús López-Herce

**Affiliations:** 1 Instituto Valenciano de Pediatría, 46004 Valencia, Spain; imanrique@rcppediatrica.org; 2 Spanish Paediatric and Neonatal Resuscitation Group, 28029 Madrid, Spain; custodiocalvomacias@gmail.com (C.C.); carrilloalvarez49@gmail.com (A.C.); vasebastian59@gmail.com (V.S.); 3 Centro de Salud Fuente de San Luis, 46013 Valencia, Spain; 4 Servicio de Cuidados Intensivos Pediátricos, Hospital General Universitario Gregorio Marañón de Madrid, Instituto de Investigación Sanitaria del Hospital Gregorio Marañón, 28007 Madrid, Spain; gema_manrique@hotmail.com; 5 Departamento de Salud Pública y Maternoinfantil, Facultad de Medicina, Universidad Complutense de Madrid, 28040 Madrid, Spain; 6 Red de Salud Maternoinfantil y del Desarrollo (RedSAMID), RETICS Financiada por el PN I+D+I 2008–2011, ISCIII—Subdirección General de Evaluación y Fomento de la Investigación y el Fondo Europeo de Desarrollo Regional (FEDER), ref. RD12/0026, 28007 Madrid, Spain

**Keywords:** cardiopulmonary resuscitation, immediate life support, resuscitation, pediatric life support

## Abstract

A retrospective analysis was performed of 1637 questionnaires among students of immediate pediatric life support (IPLS) courses. All theory and practice classes and organization and methods received an average score higher than 8.5 except for the schedule and time devoted to developing contents. All parameters evaluating instructors’ skills received a score higher than 9. Participants requested more time to practice and for course adaptation to their specific professionals needs. IPLS courses are highly valued by students. The duration of IPLS practice sessions should be increased and the course should be adapted to the specific professional needs of participants.

## 1. Introduction

Health professionals receive training in cardiopulmonary resuscitation (CPR) primarily through CPR courses. In the past, healthcare professionals attended Advanced Life Support (ALS) courses, whereas Basic Life Support (BLS) courses were offered to laypersons [1,2,3]. However, scientific societies realized that not all health professionals could attend an ALS course, and BLS courses were not always effective. 

To bridge this gap, life support courses were designed to train health professionals who are not regular members of a resuscitation team in the prevention and immediate care of patients with cardiac arrest until the arrival of a resuscitation team. The European Resuscitation Council designed Immediate Life Support (ILS) and Pediatric Immediate Life Support (PILS) courses that include lectures, skill stations, and different cardiac arrest scenarios [4].

Despite the spread of ILS courses, only a few studies have been conducted to evaluate the knowledge and skills acquired by physicians, nurses [5,6,7,8,9] and undergraduates from the Schools of Medicine [10,11] and Nursing [12,13], as well as their efficacy in reducing the mortality of cardiac arrest [14]. Indeed, a paucity of studies have been carried out to assess the quality of ILS courses [11]. Knowledge of participant’s opinions on the training received is key to assessing the quality of a course. For that purpose, this study was performed.

The main objective of our study was to assess the quality of the methodology of the PILS course using an anonymous survey of the students.

## 2. Methods

A retrospective study analyzing the anonymous questionnaire of students about the quality of the immediate pediatric life support (IPLS) courses was performed.

A PILS course was designed by the Spanish Pediatric and Neonatal Resuscitation Group (SPNRG) to train participants in the recognition and care of children with cardiac arrest, CPR measures, with special focus on initial CPR maneuvers (including BLS and AED, bag-mask ventilation, intraosseus vascular access, and drug administration), and team and work coordination. This 8–12 h PILS course was adapted to meet students’ needs, thus it occasionally included a module of basic training in initial management of trauma, neonatal CPR, or arrhythmia, depending on the center where students work. Theory sessions were either face-to-face or online via a learning platform (Table 1).

A specific survey was created by the authors to analyze the opinion of the students regarding the course (organization, methods and teacher) and each of the theoretical and practical classes. 

Once the PILS course was completed, participants were asked to answer a questionnaire anonymously. The questionnaire collected demographic data such as occupation and place of work. In addition, respondents were asked to perform an individual appraisal of each theory and practice session, course organization, methods, and instructors’ skills. Questions of the survey are included in Table 2 (occupation), Table 3 (Theory and practical sessions) and Table 4 (organization, methods and teachers). For the purposes of this study, questionnaires were distributed to evaluate IPLS courses offered via the SPNRG website between 2014 and 2019. 

Students have to evaluate each item between 0 (very poor) and 10 (excellent).

All statistical analyses were performed using the SPSS package version 20 (SPSS Inc., Chicago, IL, USA). Chi-squared test was used for comparison of categorical variables. Scores on professional categories were compared by Student’s *t*-test and ANOVA with Bonferroni’s/Games–Howell correction. A comparison between each theoretical class, practice and each item of the methodology between the different professional categories was performed. When significant differences were found, a two-by-two comparison was made using the Bonferroni adjustment if the variable had a normal distribution or Games–Howell if it did not. A *p* value < 0.05 was considered statistically significant.

## 3. Results

A total of 338 PILS courses were delivered by SPNRG-credited coordinators and instructors to 6858 participants between 1999 and 2019. We could only analyze the 1637 questionnaires corresponding to the 92.5% of the students of 80 courses in 34 different centers held between 2014 and 2019. The distribution of participants by occupation is shown in Table 2. 

### 3.1. Theory and Practice Sessions

Table 3 contains the scores obtained for theory and practice sessions and a comparison by occupation.

All theory and practice sessions obtained a mean score ≥ 8.5, with significant differences between them (*p* < 0.001). Theory sessions related to vascular access, arrhythmias, trauma and neonatal cardiopulmonary resuscitation, as well as practice sessions on vascular access, trauma and neonatal CPR were scored significantly lower (*p* < 0.001) (Table 3). General practitioners assigned significantly higher scores to most of the theory and practice sessions compared with pediatricians and nurses. 

### 3.2. Organization, Teaching Methods, and Instructors 

Table 4 shows scores for course organization, teaching methods and instructors. All parameters related to course organization and methods were given a score > 8.5, except for the schedule and time devoted to developing the contents of the course. All parameters related to the evaluation of instructors were scored 9 or higher. No significant differences were observed in the scores awarded by the different health professionals to the instructors. 

### 3.3. Comments and Suggestions

The most frequent comments and suggestions among participants were:

General comments: excellent course essential to clinical practice.

Organization: extend the duration of the course, increase the number of days and reduce the number of daily hours. Augment the duration of practice sessions so that all participants are given enough time to practice.

Materials: improve the quality of European Neonatal Pediatric Life Support guidelines; replace mannequins. 

Methods: adapt the course to nursing and primary care.

Updating: it is important to repeat the course periodically.

## 4. Discussion

Evaluating the organization, and quality of theory and practice sessions of training courses is essential to improving the quality of CPR courses. Therefore, students should not be asked to perform a general evaluation of the course, but a specific appraisal of the quality of each theory and practice session.

The number of studies performed to evaluate learning outcomes of PILS courses is extremely limited [8,9,11]. This is the first study to analyze the evaluation of a PILS course by a large sample of participants. The results obtained reveal that participants gave a high rating to the PILS course, deemed it very useful for clinical practice, and considered that it should be repeated periodically. 

Participants had a very positive opinion of the organization, methods, theory and practice sessions, and teaching abilities of instructors. However, there is room for improvement in a number of important aspects, that would enhance the quality of the course. 

In our opinion, the PILS course should be more flexible. In addition, the course should be adapted to health professionals who work in centers without advanced CPR equipment. The course should be converted from an immediate CPR course into an intermediate CPR course that includes theory–practice modules of initial neonatal CPR, trauma or arrhythmias, as has already been achieved in some of our courses. This adaptation would better meet the professional needs of participants. However, it runs the risk of becoming an advanced course or a primarily theoretical course, thereby reducing the essential time devoted to practice. In this sense, some participants, mainly nurses and primary care professionals, suggested that the course should be better adapted to their professional needs. In our opinion, with a core program, each course should be adapted to the professional profile of its participants to meet their learning needs.

The schedule and duration of the program were the worst rated parameters. Most participants deemed that the duration of the course should be extended, and that it should be divided into two days for it to be less stressing and to allow more time for practice. This may entail some challenges to course planning, but is likely to facilitate learning and practice. 

All participants remarked the relevance of increasing the time devoted to practice, with more clinical cases simulating real settings of cardiopulmonary resuscitation and team-work being necessary [15,16]. 

The theory and practice sessions of complementary modules (trauma, arrhythmia and neonatal CPR) obtained lower scores than the core modules. The reason may be that these modules were not adapted to the professional profile of participants or the difficulty in summarizing these issues, which may be very complex, in a short period of time. 

According to these comments, we have performed some changes in the structure and methodology of the PILS courses. We offer different complementary modules depending on the characteristic of the students to adapt to their professional needs (neonatal resuscitation, trauma resuscitation, arrythmia…).

In each course we adapt the schedule to the characteristics of the students and the possibilities of the organization center.

Moreover, we increased the previous online time devoted to theory, thus decreasing the presential theory time and increasing the time devoted to practice.

The study revealed some differences in the upraising of some modules based on the occupation of participants. This inconsistency could perhaps be explained by differences in the previous knowledge and skills and professional needs of participants. 

Finally, the teaching skills, coordination and attitude of instructors were scored very highly, which demonstrates the efficacy of the paediatric life support instructor courses designed by the SPNRG [17].

Our study contains some limitations. Firstly, participation in the study was voluntary and a selection bias may have occurred. However, a high proportion of participants answered the questionnaire, which suggests that the sample of participants was representative.

In order to better analyse the overall quality of the course, it would be ideal to analyse not only the opinion of the students but also their achievement results.

In addition, for this evaluation to be more comprehensive, it would be more appropriate to compare the opinions of the students with opinions of instructors and learning outcomes of participants, which was not possible due to the anonymity of the questionnaire. 

## 5. Conclusions

Evaluation questionnaires are an essential tool for evaluating the quality of training courses. PILS courses are highly rated by students both in terms of theory and practice, organization, teaching methods, and instructors’ skills. Participants highlight the need for a longer course with more time devoted to practice, and remark that the structure of the course should be adapted to their professional needs.

## Figures and Tables

**Table 1:** Pediatric Immediate Life Support Courses.

**Essential theoretical content** Introduction. Causes of cardiorespiratory arrest. Characteristics of children. Recognition of the seriously ill child. Basic CPR. Foreign body airway obstruction Airway and ventilation. Arrhythmias in children. Defibrillators. Automatic external defibrillators Vascular access, administration of liquids and medication. Management of cardiorespiratory arrest and teamwork. Paediatric resuscitation trolley
**Essential practical content** Basic infant and child CPR. Foreign body airway obstruction. (2 h). Airway and ventilation in infants and children (1 h): Oxygen therapy, aspiration, oropharyngeal cannula, mask ventilation and self-inflating bag Intraosseous access and drugs (1 h). Intermediate CPR and teamwork (2 h)
**Optional contents (according to the characteristics of the students of the course)** Practice recognizing the seriously ill child: (1 h) Diagnosis and treatment of arrhythmias: it will depend on the population to which the course is directed and whether they have a manual defibrillator or AED in their workplace. Cervical immobilization, Placement of cervical collar. Helmet removal. Supraglottic devices. Venous access. Neonatal CPR.
**Evaluation** Previous initial theory exam, online or at the beginning of the course: 10–20 multiple-choice questions. Final theory exam: 10–20 multiple-choice questions. Practical evaluation of basic infant and child CPR Practical evaluation of integrated CPR infant and child CPR Course evaluation (content, methodology and teaching staff).
**Schedule** Non presential part: Online training platform or delivery of documentation to the student with time for study. Presential part: minimum 8 h - Theory: minimum 1 h - Practice: minimum 6 h - Evaluations: minimum 1 h

**Table 2:** PILS course participants.

Occupation	Sample	Percentage
Pediatrician	361	22.0%
Intensive Care	9	2.5%
Hospital	101	28%
Resident	143	39.6%
Primary Care	102	28%
Out of hospital	5	1.4%
Unknown	1	0.3%
Nurse	1093	66.7%
Intensive Care	151	13.9%
Hospital	747	68.7%
Resident	18	1.7%
Primary Care	105	9.7%
Out of hospital	66	6.1%
Family practitioner	46	2.8%
Specialist	35	76.1%
Resident	11	23.9%
Anesthetist-adult ICU	38	2.3%
Specialist	23	60.5%
Resident	15	39.5%
Adult emergency care	46	2.8%
Hospital	44	95.7%
Out of hospital	2	4.3%
Other	51	3.1%
Unknown	2	0.1%
Total	1637	

**Table 3:** Evaluation of theory and practice sessions (mean and standard deviation).

	Global	Pediatrician	Nurse	Family Practitioner	Anesthesia-ICU	Emergency	*p*
**Theory sessions**							
Fundamentals and prevention	9.14 1.06	9.26 0.90	9.08 1.10	9.48 0.96	9.50 0.76	9.33 1.11	0.000
Basic CPR	9.23 1.04	9.31 0.89	9.19 1.07	9.63 0.79	9.34 1.25	9.37 1.08	0.003
Airway	9.12 1.12	9.15 1.03	9.11 1.14	9.61 0.68	8.71 1.37	9.15 1.26	0.008
Vascular accesses	8.87 1.37	8.98 1.31	8.86 1.35	9.39 1.04	8.84 1.22	9.07 1.56	0.000
Arrhythmias	8.46 1.75	8.85 1.43	8.26 1.88	9.15 1.36	9.11 1.11	8.96 1.69	0.000
Integrated CPR	9.15 1.20	9.33 0.96	9.06 1.29	9.63 0.74	9.45 0.89	9.33 1.07	0.000
CPR and trauma	8.54 1.76	8.65 1.67	8.43 1.83	9.29 1.16	8.90 1.09	9.03 1.40	0.037
Neonatal CPR	8.58 1.88	8.41 2.11	8.57 1.83	9.56 0.96	8.70 1.86	9.27 1.18	0.032
**Practice Sessions**							
Recognition of severe ill child	9.19 1.13	9.29 0.98	9.14 1.17	9.61 0.83	9.29 1.19	9.40 0.82	0.021
Basic CPR	9.14 1.17	9.22 1.06	9.08 1.23	9.67 0.66	9.32 0.96	9.39 0.97	0.004
Airway	9.10 1.18	9.14 1.06	9.07 1.21	9.65 0.70	8.84 1.44	9.24 1.05	0.028
Vascular accesses	8.88 1.43	8.87 1.46	8.88 1.43	9.48 0.93	8.79 1.66	9.04 1.42	0.028
Integrated CPR	9.19 1.18	9.37 0.87	9.11 1.27	9.61 0.85	9.45 0.92	9.35 0.94	0.000
CPR and trauma	8.67 1.64	8.73 1.54	8.58 1.17	9.27 1.28	8.94 1.29	9.32 1.27	0.000
Neonatal CPR	8.65 1.82	8.42 2.18	8.63 1.75	9.52 0.91	9.06 1.21	9.32 1.09	0.025

**Table 4:** Scores for teaching methods, instructors, and organization (mean and standard deviation).

	Global	Pediatrician	Nurse	Family Practitioner	Anesthesia-ICU	Emergency	*p*
**Organization**							
Organization	9.07 1.26	9.26 1.07	8.97 1.32	9.57 1.02	9.34 1.12	9.30 1.28	0.000
Classrooms	8.67 1.36	8.76 1.24	8.62 1.40	9.09 1.11	8.76 1.36	9.07 1.23	0.046
Schedule	8.30 1.71	8.46 1.62	8.20 1.75	8.70 1.16	8.58 1.50	8.85 1.73	0.015
Slides	8.91 1.27	8.99 1.20	8.87 1.30	9.37 1.04	9.05 1.18	8.98 1.80	0.116
Instructional materials	8.85 1.31	8.81 1.28	8.85 1.11	9.41 0.95	8.61 1.58	9.15 1.47	0.005
Materials distributed	8.91 1.57	8.80 1.74	8.96 1.51	9.35 1.05	8.61 1.80	9.09 1.65	0.025
**Methods**							
Methodology	9.14 1.11	9.24 0.98	9.08 1.15	9.70 0.69	9.26 0.97	9.24 1.28	0.005
Time devoted to course contents	8.43 1.67	8.70 1.51	8.29 1.73	8.89 1.28	8.92 1.21	8.89 1.16	0.000
Theory	8.96 1.20	9.05 1.10	8.90 1.24	9.43 0.88	9.08 1.12	9.09 1.34	0.022
Practice	9.17 1.14	9.31 0.98	9.10 1.21	9.46 1.13	9.42 0.91	9.37 0.92	0.005
**Teachers**							
Level of competency	9.51 0.81	9.59 0.71	9.48 0.84	9.74 0.68	9.63 0.63	9.48 0.88	0.015
Adaptation to participants	9.30 1.11	9.42 0.92	9.24 1.18	9.61 0.80	9.58 0.72	9.33 1.33	0.003
Coordination	9.38 0.98	9.45 0.85	9.35 1.03	9.65 0.87	9.50 0.68	9.54 0.80	0.049
Clarity of expression	9.43 0.91	9.51 0.82	9.40 0.94	9.67 0.70	9.45 0.76	9.52 0.93	0.002
Ability to raise interest	9.43 0.92	9.50 0.77	9.39 0.98	9.74 0.71	9.53 0.76	9.54 1.00	0.007
Ability to make corrections in a constructive manner	9.35 1.08	9.45 0.94	9.31 1.14	9.70 0.72	9.45 0.89	9.48 1.22	0.031
